# The outcomes of total knee arthroplasty in morbidly obese patients: a systematic review of the literature

**DOI:** 10.1007/s00402-019-03127-5

**Published:** 2019-02-16

**Authors:** Louis Boyce, Anoop Prasad, Matthew Barrett, Sebastian Dawson-Bowling, Steven Millington, Sammy A. Hanna, Pramod Achan

**Affiliations:** 1 Barts and the London School of Medicine and Dentistry, Whitechapel, London, E1 2AD UK; 2 Royal London Hospital, Barts Health NHS Trust, Whitechapel, London, E1 1BB UK

**Keywords:** Total knee arthroplasty, TKA, Total knee replacement, TKR, Morbid obesity, Obesity, Non-obese, Revision rate, Knee Society Objective scores, KSOS, Knee Society Functional scores, KSFS, Complications, Complication rates, Infection, Infection rates, Prosthetic infection, Superficial wound infection, Wound healing problems, Wound healing delay, Quality of life, Functional outcome

## Abstract

**Introduction:**

The increasing prevalence of obesity has led to an increase in total knee arthroplasties (TKAs) being undertaken in patients with a higher body mass index (BMI). TKA in morbidly obese patients can be technically challenging due to numerous anatomical factors and patient co-morbidities. The long-term outcomes in this patient group are unclear. This systematic review aims to compare the long-term revision rates, functional outcomes and complication rates of TKAs in morbidly obese versus non-obese patients.

**Methods:**

A search of PubMed, EMBASE and PubMed Central was conducted to identify studies that reported revision rates in a cohort of morbidly obese patients (BMI ≥ 40 kg/m^2^) that underwent primary TKA, compared to non-obese patients (BMI ≤ 30 kg/m^2^). Secondary outcomes included Knee Society Objective Scores (KSOS), Knee Society Functional Scores (KSFS), and complication rates between the two groups. The difference in revision rates was assessed using the Chi-squared test. The Wilcoxon signed-rank test was used to compare pre-operative and post-operative functional scores for each group. KSOS and KSFS for morbidly obese and non-obese patients were compared using the Mann–Whitney test. Statistical significance was defined as *p* ≤ 0.05.

**Results:**

Nine studies were included in this review. There were 624 TKAs in morbidly obese patients and 9,449 TKAs in non-obese patients, average BMI values were 45.0 kg/m^2^ (range 40–66 kg/m^2^) and 26.5 kg/m^2^ (range 11–30 kg/m^2^) respectively. The average follow-up time was 4.8 years (range 0.5–14.1) and 5.2 years (range 0.5–13.2) respectively, with a revision rate of 7% and 2% (*p* < 0.001) respectively. All functional scores improved after TKA (*p* < 0.001). Pre- and post-operative KSOS and KSFS were poorer in morbidly obese patients, however, mean improvement in KSOS was the same in both groups and comparable between groups for KSFS (*p* = 0.78). Overall complication rates,  including infection, were higher in morbidly obese patients.

**Conclusions:**

This review suggests an increased mid to long-term revision rate following primary TKA in morbidly obese patients, however, these patients have a functional recovery which is comparable to non-obese individuals. There is also an increased risk of perioperative complications, such as superficial wound infection. Morbidly obese patients should be fully informed of these issues prior to undergoing primary TKA.

## Introduction

Total knee arthroplasty (TKA) is one of the most commonly performed orthopaedic procedures in the UK with 108,713 TKAs carried out in 2016 [1]. This number is expected to rise to 118,666 by 2035 [2]. The prevalence of obesity is also increasing, with UK trends predicting a rise from 26% in 2008 to 41–48% in men and 35–43% in women by 2035 [3]. As obesity is a risk factor for osteoarthritis (OA), especially in the knee [4], the increase in prevalence has led to an increased number of TKAs being performed on obese patients [5]. In the UK, obese patients comprised 56% of primary TKAs in the 2016 [1]. Whilst the exact mechanism is not known, excessive joint loading in obese patients is thought to alter gait and movement strategies, resulting in joint malalignment and cartilage degeneration [6]. In addition, obesity-related dyslipidaemia has been shown to induce joint damage through the actions of pro-inflammatory adipokines and cytokines [7].

The long-term outcome of TKA in obese patients remains a debated issue. Whilst some studies have shown favourable results [8, 9], others have not [10, 11]. Studies attempting to compare the outcome of primary TKA in obese versus non-obese patients have also shown mixed results [10, 12–16]. Some studies reported increased revision rates, lower functional scores and increased complication rates, including infection [10, 12, 15], whilst others failed to demonstrate any significant difference [13, 14, 16]. This lack of evidence has influenced recent policy-making within the National Health Service (NHS) in the United Kingdom, with some Clinical Commissioning Groups (CCGs) imposing restrictions on offering TKA to obese patients [17].

This systematic review aims to compare the long-term outcomes of TKA in morbidly obese versus non-obese patients. The primary outcome measure was the revision rate and secondary measures included the functional outcome and incidence of complications.

## Method

### Search strategy

The Preferred Reporting Items for Systematic Reviews and Meta-Analyses (PRISMA) guidelines were followed [18]. An electronic database search of PubMed, EMBASE and PubMed Central was conducted, to search for studies reporting revision rates of TKAs in morbidly obese patients. The following search string was used: “(total knee replacement OR total knee arthroplasty) AND morbid obesity”. This search returned relevant studies published between the time of inception of the databases to June 2017.

### Eligibility criteria

The inclusion criterion was agreed upon by authors LB, AP and SH prior to the identification phase. Studies were included that reported revision rate in morbidly obese patients (BMI > 40 kg/m^2^) who had undergone primary TKA versus a non-obese group. Studies with a mean follow-up period of less than 2 years and those not published in English were excluded. Studies that did not directly report revision rates were included if they provided sufficient data from which revision rate could be calculated.

### Data extraction

Screening was performed in three phases to identify relevant titles, abstracts and full texts. Two reviewers (LB, AP) extracted the data through a standardized data collection form. Three reviewers (LB, AP, SH) checked the data for accuracy and any inconsistent results were handled by discussion. The following data: number of patients, number of knees, revision rates, overall complication rates, rates of superficial wound infection, prosthetic joint infection and wound healing problems, mean pre- and postoperative Knee Society scores (KSS) and the mean and range for BMI, age and follow-up were extracted.

### Statistical analysis

Non-parametric tests were used for statistical analyses. The significance of the difference between revision rates in the morbidly obese and non-obese groups was calculated using the Chi-squared test. The Wilcoxon signed-rank test was used to compare pre-operative and post-operative functional scores for each group separately. The Mann–Whitney test was used to compare pre-operative morbidly obese and non-obese functional scores and post-operative morbidly obese and non-obese functional scores. Statistical significance was defined as *p* ≤ 0.05.

## Results

### Search results

**Fig. 1:**
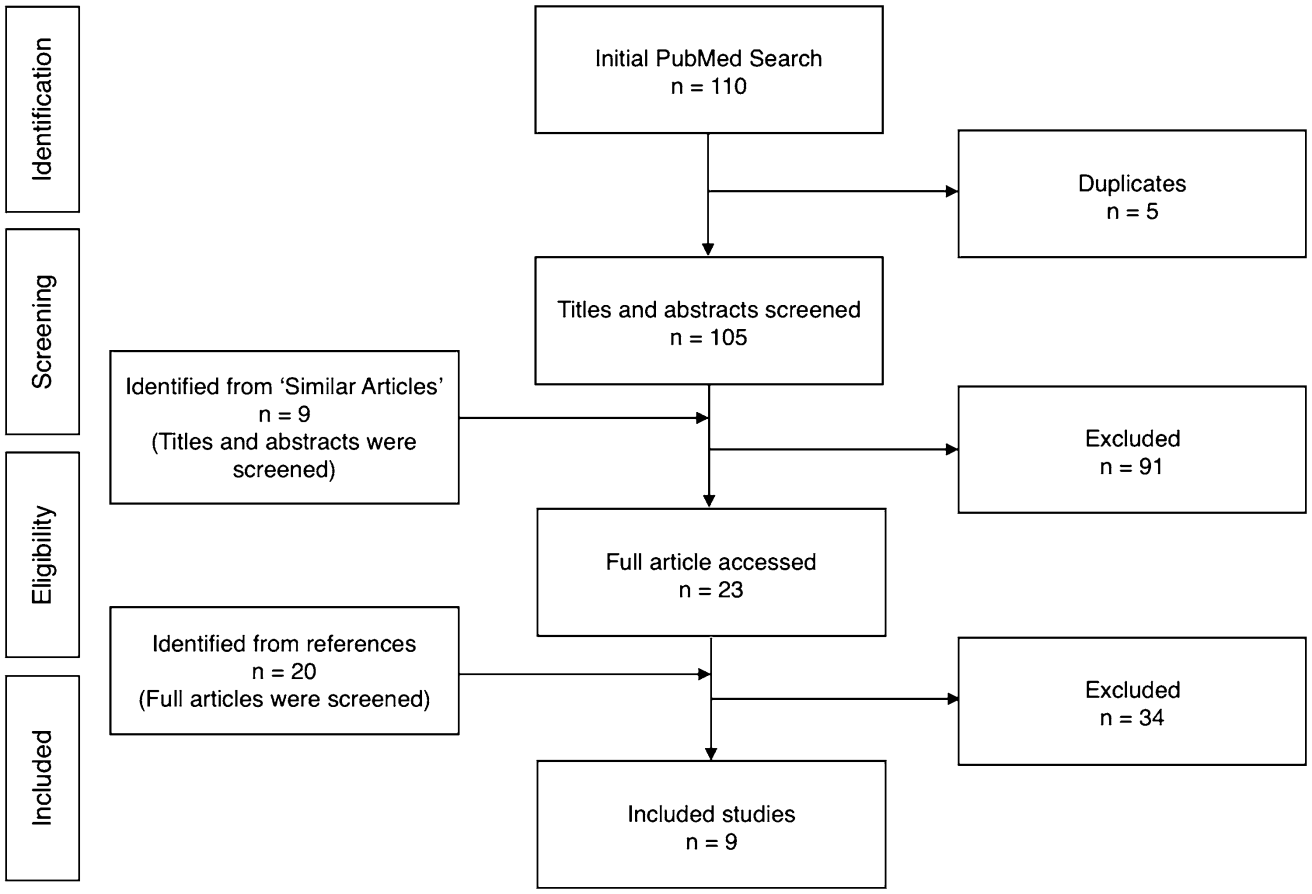
Preferred Reporting Items for Systematic Reviews and Meta-Analyses (PRISMA) flow chart for showing the identification of the included studies

The PRISMA flowchart for study selection is shown in Fig. 1. The initial PubMed search returned 110 abstracts which were screened for eligibility. After removal of duplicates, and studies that did not fit our eligibility criteria, nine studies were included for review [15, 19–26].

### Cohort characteristics

Patient demographics for each study are summarised in Table 1. The total reported number of knees in the morbidly obese and non-obese groups across all studies was 624 and 9449, respectively. The mean BMI for morbidly obese patients was 45.0 kg/m^2^ (range 40–66 kg/m^2^) and 26.5 kg/m^2^ (range 11–30 kg/m^2^) for non-obese patients. The average morbidly obese patient underwent their TKA at a younger age (62.9 years) than the average non-obese patient (66.2 years). Bordini et al. only provided a mean age for all patients (72 years) [19]. The most common indication for surgery was osteoarthritis. The average follow-up time was 4.8 years (range 0.5–14.1 years) in the morbidly obese and 5.2 years (range 0.5–13.2 years) in non-obese patients (Table 2). Naziri et al. did not report mean follow-up time in the control group but stated that the follow-up times were matched with the morbidly obese patients within four months [24]. Bordini et al. only provided a mean follow-up time for all patients (3.1 years) [19].

**Table 1:** Mean BMI (and range) and mean age (and range) for morbidly obese (MO) and non-obese (NO) patients in each study

Study	Year	Mean BMI, kg/m^2^ (range)		Mean age, years (range)	
		MO	NO	MO	NO
Spicer et al. [24]	2001	NR (> 40)	NR (< 30)	63 (41–78)	70 (35–83)
Foran et al. [21]	2004	43 (40–47)	26 (18–30)	65 (32–84)	70 (42–84)
Amin et al. [14]	2006	43 (40–61)	27 (23–30)	62 (40–80)	63 (42–80)
Ersozlu et al. [20]	2007	42 (40–45)	27 (24–30)	60 (NR)	67 (NR)
Krushell et al. [22]	2007	44 (40–53)	26 (20–29)	67 (48–81)	69 (39–82)
Bordini et al. [18]	2009	NR (> 40)	NR (< 30)	72 (71.8–72.1)	72 (71.8–72.1)
Dewan et al. [25]	2009	44 (> 40)	25 (20–29)	58 (NR)	66 (NR)
Naziri et al. [23]	2013	54 (50–66)	28 (25–30)	60 (43–74)	59 (45–75)
Chen et al. [19]	2016	NR (> 40)	NR (< 30)	61 (NR)	68 (NR)

*NR* not reported

**Table 2:** Number of patients, number of knees, mean follow-up time (and range) and revision rate for morbidly obese (MO) and non-obese (NO) patients in each study

Study	Year	Patients (knees)		Mean follow-up, years (range)		Revision rate, %		*p* value
		MO	NO	MO	NO	MO	NO	
Spicer et al. [24]	2001	NR (59)	371 (425)	6.1 (4–12)	6.3 (4–12)	5	3	NR
Foran et al. [21]	2004	11 (12)	68 (78)	6.6 (5–8.9)	6.9 (5–10.3)	8	0	0.02
Amin et al. [14]	2006	38 (41)	38 (41)	3.2 (0.5–5.5)	3.7 (0.5–5.6)	26	0	0.01
Ersozlu et al. [20]	2007	21 (42)	20 (40)	2.7 (2–3.3)	2.7 (2–3.3)	0	0	NR
Krushell et al. [22]	2007	NR (39)	NR (39)	7.5 (5.2–14.1)	7.5 (5–13.2)	5	0	NR
Bordini et al. [18]	2009	NR (172)	NR (6532)	3.1 (1.5–6)	3.1 (1.5–6)	2	2	NR
Dewan et al. [25]	2009	31 (41)	67 (85)	4 (NR)	6 (NR)	7	5	0.816
Naziri et al. [23]	2013	95 (101)	95 (101)	5.2 (3–7.1)	NR	7	3	0.28
Chen et al. [19]	2016	117 (117)	2108 (2108)	NR (2–10)	NR (2–10)	2	1	0.703

*NR* not reported

### Outcome analysis

#### Revision rate

The mean revision rates were 7% in the morbidly obese and 2% in non-obese patients (*p* < 0.001) (Table 2). Two studies reported revision rates directly [21, 26], while in eight studies [15, 19, 20, 22–24] the rates were calculated as the percentage of knees that underwent revision during follow-up.

#### Functional scores

Knee Society Objective Scores (KSOS) are shown in Table 3 and Knee Society Function scores (KSFS) in Table 4. Seven studies reported KSOS and KSFS [15, 20, 21, 23–26]. All functional scores showed a significant improvement after TKA (*p* < 0.001). Mean preoperative KSOS were 43 (range 0–78) and 47 (range 0–83) (*p* = 0.65) and mean postoperative KSOS were 87 (range 32–100) and 91 (range 45–100) (*p* = 0.04) in morbidly and non-obese patients, respectively. Mean improvement in KSOS was 44 in both morbidly and non-obese groups. Mean preoperative KSFS were 40 (range 0–85) and 47 (range 0–97) (*p* = 0.20), mean postoperative KSFS were 67 (range 0–100) and 76 (range 20–100) (*p* = 0.20) and mean improvement in scores were 27 and 29 (*p* = 0.78) in morbidly and non-obese patients, respectively.

**Table 3:** Mean pre- and postoperative Knee Society Objective scores (KSOS) and improvement in KSOS for morbidly obese (MO) and non-obese (NO) patients in each study

Study	Year	Mean preoperative KSOS (range)		*p* value	Mean postoperative KSOS (range)		*p* value	Mean improvement in KSOS		*p* value
		MO	NO		MO			MO	NO	
Spicer et al. [24]	2001	45 (NR)	48 (NR)	NR	86 (NR)	91 (NR)	NR	41	43	NR
Amin et al. [14]	2006	28 (0–57)	30 (0–56)	0.5	86 (32–97)	91 (45–100)	0.08	58	61	NR
Ersozlu et al. [20]	2007	61 (42–76)	70 (61–83)	NR	87 (57–94)	91 (64–97)	NR	26	21	NR
Krushell et al. [22]	2007	30 (14–65)	34 (13–70)	NR	91 (50–100)	94 (50–100)	NR	61	60	NR
Dewan et al. [25]	2009	53 (NR)	55 (NR)	0.737	85 (NR)	89 (NR)	0.244	32	34	NR
Naziri et al. [23]	2013	53 (23–78)	50 (35–69)	0.0899	91 (58–100)	94 (66–100)	0.1161	42	44	NR
Chen et al. [19]	2016	33 (30–36)	40 (39–40)	< 0.001	83 (81–85)	85 (84–85)	0.013	50	45	0.003

*NR* not reported

**Table 4:** Mean pre- and postoperative Knee Society Function scores (KSFS) and improvement in KSFS for morbidly obese (MO) and non-obese (NO) patients in each study

Study	Year	Mean preoperative KSFS (range)		*p* value	Mean postoperative KSFS (range)		*p* value	Mean improvement in KSFS		*p* value
		MO	NO		MO			MO	NO	
Spicer et al. [24]	2001	20 (NR)	30 (NR)	0.003	60 (NR)	68 (NR)	NR	40	38	NR
Amin et al. [14]	2006	51 (0–75)	52 (10–80)	0.5	76 (30–100)	83 (35–100)	0.01	25	31	NR
Ersozlu et al. [20]	2007	46 (39–74)	56 (64–97)	NR	80 (55–83)	86 (60–100)	NR	46	30	NR
Krushell et al. [22]	2007	31 (0–50)	38 (0–80)	NR	44 (0–90)	64 (20–100)	< 0.005	13	26	NR
Dewan et al. [25]	2009	42 (NR)	46 (NR)	0.119	68 (NR)	66 (NR)	0.313	26	20	NR
Naziri et al. [23]	2013	52 (0–85)	54 (35–70)	0.1589	82 (30–100)	90 (64–100)	0.004	30	36	NR
Chen et al. [19]	2016	39 (36–42)	53 (52–54)	< 0.001	58 (55–62)	74 (73–75)	< 0.001	19	21	0.736

*NR* not reported

#### Complication rates

Morbidly obese patients had higher overall complication rates, and higher rates of superficial wound infection, prosthetic joint infection, wound healing problems or delay compared to non-obese patients in all studies (Table 5). Four studies reported overall complication rates [15, 21, 24, 26], four studies reported superficial wound infections [15, 19, 21, 24], two studies reported prosthetic joint infections [15, 21], two studies reported wound healing problems or delay [23, 24]. Dewan et al. only reported infection rates [26]. Morbidly obese patients also had higher rates of aseptic and radiographic loosening, and osteolysis or wear (Table 5). Three studies reported aseptic loosening [15, 23, 24], two studies reported radiographic loosening [15, 21], one study reported osteolysis or wear [23].

**Table 5:** Complication rates for morbidly obese (MO) and non-obese (NO) patients in each study

Study	Year	Complication rates	
		MO	NO
Amin et al. [14]	2006	Overall complication rate: 32%	Overall complication rate: 0%
		Superficial wound infections: 17%	Superficial wound infections: 0%
		Prosthetic joint infections: 10%	Prosthetic joint infections: 0%
		Radiographic loosening: 4.9%	Radiographic loosening: 0%
		Aseptic loosening: 9.8%	Aseptic loosening: 0%
Ersozlu et al. [20]	2007	Overall complication rate: 30%	Overall complication rate: 25%
		Superficial wound infections: 19%	Superficial wound infections: 5%
		Prosthetic joint infections: 0%	Prosthetic joint infections: 0%
		Radiographic loosening: 0%	Radiographic loosening: 0%
Krushell et al. [22]	2007	Wound-healing problems: 20.5%	Wound-healing problems: 0%
		Aseptic loosening: 2.6%	Aseptic loosening: 0%
		Osteolysis or wear: 2.6%	Osteolysis or wear: 0%
Bordini et al. [18]	2009	Superficial wound infections: 0%	Superficial wound infections: 0%
Dewan et al. [25]	2009	Overall complication rate: 26%	Overall complication: 15%
		Infection: 7%	Infection: 4%
Naziri et al. [23]	2013	Overall complication rate: 14%	Overall complication rate: 5%
		Superficial wound infection: 1%	Aseptic loosening: 0%
		Delayed wound healing: 1%	
		Aseptic loosening: 4%	

## Discussion

This review has shown that morbidly obese TKA patient have significantly higher revision rates (*p* < 0.001) and greater complication rates, including higher rates of infection and wound healing problems, compared with their non-obese counterparts. However, morbidly obese and non-obese patients experience similar improvements in KSOS and KSFS after TKA. All patients undergoing TKA benefit to the same extent from improvements in knee-related function and quality of life, regardless of BMI.

Global obesity trends predict 20% of the world adult population could be obese by 2030, equating to 1.12 billion individuals. In high-income OECD countries, including the UK, US, France and Germany, 37% of adults are expected to be obese by 2030 [27]. Obesity is a risk factor for osteoarthritis and contributes to the demand for TKA in these patients. These projections indicate that an increasing number of morbidly obese patients will warrant TKA in the future.

Morbid obesity has been widely reported to increase the risk of perioperative complications during TKA, including superficial wound infections and prosthetic joint infections [12, 28, 29]. Whilst the exact mechanism is unclear, this may be partly explained by a weakened immune response in obese patients. The number of monocytes that mature to macrophages was found to be significantly less in obese patients [30]. Impaired release of lymphocyte migration-inhibiting factor has also been found in insulin-resistant, non-ketotic diabetic and non-hyperglycaemic obese patients [31]. Furthermore, obesity is strongly associated with reduced subcutaneous tissue oxygenation, which is in turn linked to higher rates of infection [32]. Our review finds a higher incidence of complications in morbidly obese patients. Amin et al. found the greatest difference in complication rate [15]. No complications were reported in their control group, while 32% of morbidly obese knees experienced complications; 17% were superficial wound infections, and 5% were prosthetic joint infections [15]. This may in part be due to surgery in morbidly obese patients being more technically demanding, resulting in longer operative time which increases the risk of postoperative infections [33, 34].

The association between BMI and the long-term outcome of TKA is unclear. Studies have shown increased revision rates and lower functional scores in obese patients [35–37] whilst other studies have reported similar outcomes regardless of BMI [9, 38, 39]. Gaillard et al. found that obesity did not affect mid-term implant survival, though their results indicated poorer functional outcomes and a risk of postoperative complications in obese patients [40]. This review suggests that revision rates are greater in morbidly obese patients. Amin et al. reported the greatest difference in revision rates between morbidly obese and non-obese patients, where survivorship rates were 26% and 0%, respectively [15]. A common assumption is that overloading of the knee occurs in patients with high BMI, resulting in greater impact loading across the tibial component, therefore, increased component loosening and poorer implant survival [15, 24, 41]. In spite of this, it has been suggested that a more sedentary lifestyle in morbidly obese patients counterbalances the increased rate of prosthesis wear, which may explain the small difference in revision rate reported in the review [15, 42]. Patient-specific guide technology has also been shown to reliably correct mechanical alignment in obese patients without adversely affecting outcomes [43].

Mean pre- and postoperative overall KSOS and KSFS were consistently lower in morbidly obese patients, though, and perhaps more importantly, the mean score improvements were comparable between the two groups. Chen et al. studied the largest number of patients, 117 morbidly obese and 2108 non-obese patients, and found comparable KSFS between the two groups as well as superior improvements in KSOS [20]. The authors of this study suggest sample sizes in other studies are too small to detect true differences in functional scores. Krushell et al. reported the poorest mean improvement in KSFS of 13 in the morbidly obese group versus 26 in the non-obese group [23]. Though this improvement may seem insignificant numerically, it is approximately equivalent to being housebound preoperatively, and able to walk 400 metres postoperatively [44]. These findings suggest TKA offers substantial benefits to morbidly obese patients in terms of pain relief, knee stability, range of movement, walking distance and climbing stairs.

Limitations to this review include the large range in BMI within both study groups and the small sample sizes. Follow-up times ranged from 0.5 to 14.1 years, therefore, making it difficult to compare short, medium and long-term implant survivorship. Other confounding variables included comorbidities, prosthesis type, population heterogeneity, activity level, laterality and surgical technique. Individual ages were not reported in the included studies, therefore, statistical significance could not be calculated. Six studies were retrospective, and therefore, susceptible to selection bias [19–21, 23, 24, 35]. Our statistical analyses were limited to non-parametric methods, because a normal distribution could not be assumed from the reported data. More randomised controlled trials and prospective cohort studies are required to assess long-term outcomes in morbidly obese patients.

## Conclusion

This review indicates that revision rate of TKA in morbidly obese individuals is increased compared with non-obese patients [7% vs. 2% (*p* < 0.001)]. There is also an increased risk of perioperative complications, mainly superficial wound infections. Nevertheless, all patients regardless of BMI experience comparable improvements in knee function. In conclusion, obese patients should be counselled regarding the increased risk of failure and inferior functional outcome, and should be encouraged to lose weight prior to undergoing TKA. These patients, however, should not be refused TKA based on their BMI value alone, as the procedure is likely to offer them a significant improvement in functional outcome and quality of life.
